# Leptomeningitis as Rare Secondary Dissemination in MEITL (Monomorphic Intestinal Epitheliotropic T-Cell Lymphoma)

**DOI:** 10.3390/life15081243

**Published:** 2025-08-05

**Authors:** Mihaiela Lungu, Violeta Diana Oprea, Elena Niculeț, Luminița Lăcrămioara Apostol, Marius Ionuț Păduraru, Ana Maria Ionescu, Andrei Lucian Zaharia

**Affiliations:** 1 Clinical-Medical Department, Faculty of Medicine and Pharmacy, “Lower Danube” University of Galați, 800008 Galați, Romania; micalungu@gmail.com (M.L.); lum.apostol@gmail.com (L.L.A.); zaharia.andreilucian@gmail.com (A.L.Z.); 2 Medical Department, Sjællands Universitetshospital Nykøbing Falster, 4800 Nykøbing Falster, Denmark; 3 Department of Morphological and Functional Sciences, Faculty of Medicine and Pharmacy, “Lower Danube” University of Galați, 800008 Galați, Romania; helena_badiu@yahoo.com; 4 Medical Department, Faculty of Medicine and Pharmacy, “Lower Danube” University of Galați, 800201 Galați, Romania; pad_marius89@yahoo.com; 5 Department of Neurology, Faculty of Medicine, Ovidius University of Constanța, 900527 Constanța, Romania; iuliusana@gmail.com

**Keywords:** leptomeningitis, monomorphic intestinal epitheliotropic T-cell lymphoma (MEITL), Type II enteropathy-associated T-cell lymphoma (EATL)

## Abstract

(1) Background: Monomorphic intestinal epitheliotropic T-cell lymphoma (MEITL) is a very rare subtype of lymphoma, being involved in less than 5% of lymphomas of the digestive tract. Accurate diagnosis is extremely challenging due to the lack of specific clinical symptoms and the low specificity of the diagnostic approaches. (2) Methods: We present the case of a patient admitted to the Neurology Clinic of the Emergency Clinical Hospital of Galati, Romania, with progressive cranial nerve impairment. (3) Results: Analyzing clinical and paraclinical data and corroborating the previous known diagnosis of MEITL, the positive diagnosis was that of meningitis with atypical lymphocytes with MEITL as starting point. The cytology of CSF was the basis for the diagnostic confirmation. (4) Conclusions: The present case is a rare situation of secondary dissemination of MEITL. We were not able to identify a similar report in the available literature that associated urothelial carcinoma with leptomeningeal MEITL-sourced neoplastic lesions.

## 1. Introduction

Monomorphic intestinal epitheliotropic T-cell lymphoma (MEITL), a primary intestinal lymphoma, is a rare, aggressive malignant tumor found more commonly in Asians and Hispanics without a history of celiac disease. It is part of the non-Hodgkin’s T-cell lymphomas, being initially referred to as EATL—Type II enteropathy-associated T-cell lymphoma [1]. Since 2017, it has been recognized by WHO as MEITL. It mainly affects the male population, and in 83–90% of cases it involves the small intestine; the colon, duodenum, or stomach are seldom affected [2]. The tumor cells often display an activated cytotoxic T-cell phenotype and express CD8 and CD56.

Clinically, patients may experience abdominal pain, anorexia, intestinal perforation, weight loss, or diarrhea. Clinical examination usually reveals a small tumor mass in the small intestine, or regional adenopathy [3].

Due to unspecific digestive or general symptoms, most patients are diagnosed in the late stages of disease (stage IV) and by pathological examination the tumor is classified into two categories: typical MEITL (58% of cases) and atypical MEITL (42% of cases), the latter of which is non-monomorphic, with necrosis, has an angiotrophic or starry-sky pattern, and a homogeneous immunophenotypic profile [4,5]. A comprehensive clinical, pathological, and genomic study of 71 European MEITL patients [4] showed that the analyzed tumors shared a homogeneous immunophenotypic profile, expressing surface activation markers in various proportions (CD3+ for 98%, CD4− for 94%, CD5− for 97%, CD7+ for 97%, CD8+ for 90%, CD56+ for 86%, CD103+ for 80%, and cytotoxic marker+ for 98%) with more frequent expression of TCRγδ (50%) than TCRαβ (32%) [4].

In total, 97% of MEITL patients have deletion-type mutations on the SETD2 gene, and less often on STAT5B, JAK3, TP53, JAK1, BCOR, ATM [6,7,8,9]. In the common type, medium-sized monomorphic cells with round nuclei and pale cytoplasm are described, infiltrating the intestinal epithelium and lacking significant necrosis or inflammation [2].

As a rare condition, there is still no well-defined therapeutic protocol, but surgery, radiotherapy and chemotherapy with cyclophosphamide, and doxorubicin or vincristine can be beneficial. Stem cell transplant procedures are also an option.

MEITL is an aggressive neoplasia, resistant to conventional therapies and characterized by driver gene alterations dysregulating histone methylation, JAK/STAT signaling and encompassing genetic and morphologic variants associated with very high risk [8].

The early clinical symptoms of MEITL are insidious and can manifest as nonspecific intestinal symptoms. Therefore, most MEITLs are found at an advanced stage with an extremely poor prognosis: the median survival time of MEITLs is only 7 months, and the 1-year overall survival rate is only 36%.

The median survival time from diagnosis is approximately 7–12 months in patients treated with anthracycline [3]. A recent study of 71 European MEITL patients revealed a broad morphological variation with only 58% typical cases, while the remaining 42% had atypical morphological features [4].

Cerebral metastasis of MEITL appears as a rare clinical situation; few cases are cited in the literature and none have been identified at the level of the leptomeninges [10].

## 2. Materials and Methods

We present the case of a patient admitted to the Neurology Clinic of the Emergency Clinical Hospital of Galati, Romania, for progressive facial paresis due to VII cranial nerve impairment.

## 3. Results

We present the case of a 75-year-old patient, who suffered a laparotomy in June 2024 related to peritonitis, which developed due to a perforated intestinal tumor. The histopathological examination established the diagnosis of MEITL. At the intestinal surgery moment, on gross examination, the specimen was described as a perforated 8.5/5 cm small intestine fragment with a tumor mass located at 2 cm from the surgical resection margin. The solid mass appeared whitish brown, with circumferential extension involving 2.5 cm of intestinal length.

Upon microscopic evaluation, the tumor mass revealed a diffuse proliferation of atypical lymphocytes with increased mitotic activity (including atypical mitoses) and ulceration of the resected specimen, involving tunica serosa of the intestinal wall, with associated lymphoepithelial lesions, hemorrhagic areas, and acute serosal inflammatory exudate (peritonitis). The surgical sample presented the specific aspect of epitheliotropism, with infiltration of the neoplastic cells into the intestinal epithelium, creating a distortion of the villous architecture. The microscopic examination confirmed the presence of specific cellularity for MEITL, neoplastic T-lymphocytes appearing medium-sized and uniform in size and shape. Nuclei with finely dispersed chromatin of a mostly round shape were present, while the cytoplasm presented as slightly eosinophilic. Immunohistochemistry identified T-cell positivity for CD3, CD7, CD8, and TIA-1.

At the same time, the patient also presented a bladder mass (subsequently removed), which was described by microscopic pathology examination as a well differentiated (G1) invasive papillary urothelial carcinoma. Postoperatively, the patient did not agree to follow any oncological treatment.

Four months after the abdominal procedure, the patient’s admission to our Neurology Department occurred following a progressive cranial nerve impairment: on the right-side, VII, IX, X cranial nerves were involved, while on the left side, III, VII, IX, X cranial nerves were affected, resulting in severe dysphagia, associated with an ALS-type syndrome and amyotrophy of the interosseous muscles of the hands, as another associated paraneoplastic symptom.

The patient’s history revealed the recent MEITL (intestinal tumor diagnosed in June 2024 after surgical resection) and bladder carcinoma (urothelial carcinoma diagnosed and treated with chemotherapy, later in 2024).

Anamnesis did not show relevant medical history or comorbidities. The patient reported no family history of malignant tumors.

The involvement of dysfunctions of cranial nerves III–XI progressively evolved in the last month prior to admission. By correlating the results of the patient’s MRI gadolinium contrast examination with the CSF cytology examination and the personal medical history, the diagnosis of meningitis secondary to MEITL was established.

Only whole brain radiotherapy was used, as the patient refused other available therapeutic options, i.e., chemotherapy.

Laboratory findings revealed an important inflammatory syndrome, with increased levels of C-reactive protein, fibrinogen, and ESR.

**Figure 1:**
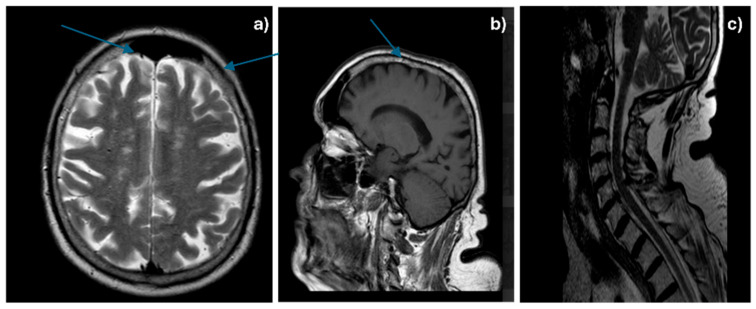
Cerebral (**a**,**b**—arrows showing diffuse meningeal thickening) and cervical (**c**) gadolinophilic leptomeningeal infiltrate, associated with vascular-degenerative lesions.

Considering the patient’s medical history, an MRI with gadolinium contrast was performed, which revealed a diffuse thickening of the meninges at the cerebral and cervical level, enhanced with contrast and associated with vascular-degenerative lesions located in the white matter—Figure 1a–c.

Considering the patient’s particular medical background of suffering from two synchronous tumors, a secondary determination was strongly suggested, so a biochemical and cytological examination of the CSF was performed to determine the type of tumor cells that infiltrated the meninges. Table 1 shows relevant CSF biochemical analysis findings. These results support the clinical suspicion of neoplastic meningitis but are not diagnostic on their own. A definitive diagnosis requires identification of malignant cells in the CSF through cytology and/or flow cytometry.

**Figure 2:**
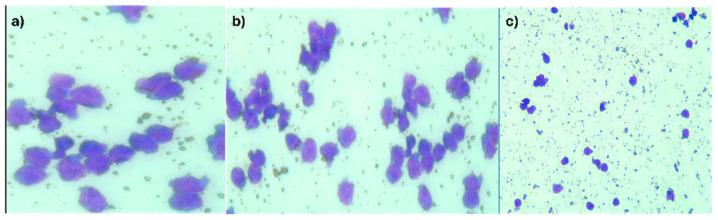
(**a**–**c**) CSF with atypical monomorphic lymphocyte infiltrate and cellular debris. (May-Grunwald Giemsa (MGG) staining: (**a**) MGG × 400; (**b**) MGG × 200; (**c**) MGG × 100).

The cytology examination of the CSF described a smear with atypical, monomorphous lymphocytes, without any malignant transitional cells that could have been indicating urothelial carcinoma)—Figure 2a–c.

A flow cytometric analysis of CSF was unfortunately not available to clearly confirm the positive diagnosis. The positive diagnosis based on CSF findings, corroborated by the recent MEITL history where the description of CSF lymphocytes matched the primary tumor cells.

The patient was redirected to the radiotherapy service, where 3 DCRT in total dose = 20 Gy/5 fr/10 days, D/fr = 400 cGy/PT-whole brain were administered. Radiotherapy was well tolerated and the patient remained in oncological and urological care.

## 4. Discussion

MEITL associated with urothelial carcinoma and meningeal neoplastic dissemination is a clinical situation not yet found in the available medical literature.

Secondary cerebral dissemination in MEITL is considered a very rare situation that targets the following regions: supratentorial (subcortical and periventricular region) [11,12], infratentorial (cerebellum)-supratentorial [13,14], infratentorial (cerebellum) [13,14], supratentorial with involvement of the frontal and parietal lobes, and frontal and corpus callosum [11]. Depending on the location of the secondary cerebral dissemination, patients may suffer from headache, depression, confusion, memory loss, ataxia, aphasia, epileptic seizures, and motor deficits [15,16,17].

Chuah et al. [11] describes a group of five patients with MEITL, 83% of whom had supratentorial brain metastases and only one case (17%) with a cerebellar location.

In the medical literature, secondary meningeal dissemination was described by Uematsu et al. [12] and Yuma Nato et al. [10], considered a rather rare occurrence. Yuma Nato et al. described a case of early central nervous system relapses of MEITL in a patient who underwent a heart transplant procedure, an event occurring 7 months after the cardiac procedure and manifesting with the progressive loss of cognitive abilities due to leptomeningitis.

Meningitis in the context of MEITL is a rare but possible extra-gastrointestinal dissemination, taking the hematogenous route or by direct extension, especially in advanced, recurrent cases [13,14,15,16].

Its diagnosis requires CSF cytology examination, as well as MRI examination with a contrast agent. As the treatment choice for leptomeningitis, focal brain radiotherapy or intrathecal chemotherapy with methotrexate or cytarabine may be optimal [13,14,15,16].

The inflammatory biological syndrome, presence of the intestinal tumor with specific histopathology, and the CSF appearance, all contributed to the positive diagnosis [18]. The cytological aspect of the CSF brought us to the diagnosis MEITL meningitis, as the atypical lymphocytes were intermediate in size, with round or irregular contours and inconspicuous nucleoli (as opposed to a primary central nervous system lymphoma which presents with intermediate to large cells, abnormal chromatin, prominent nucleoli and reactive background lymphocytes—aspects which were discordant with our findings) [19,20].

Flow cytometry can be a valuable tool in diagnosing leptomeningeal disease, especially in a multiparameter analysis, which can provide detailed information about CSF cell populations and improve diagnostic accuracy. However, although we are an established medical university center, this method is not available in our center. Also, upon assessment of diagnosis in this particular case, the clinical team evaluated the available guidelines and found a lack of standardized recommendations with regard to preparing the samples, panel of antibodies and even in interpretation criteria, which are not widely consistent throughout the various guidelines.

In the neurological practice of the Emergency Clinical Hospital of Galati, Romania, we have also encountered a case of NK/T-cell lymphoma with extremely rare dissemination, namely at the orbital level [18].

In the case of our presented patient, there was no secondary dissemination to other organs, although MEITL can also metastasize to the lymph nodes, liver or lung.

The positive diagnosis of secondary leptomeningeal carcinomatosis can be challenging without CSF flow cytometry, which is highly sensitive for detecting malignant cells. However, a diagnosis can still be made based on a combination of clinical presentation, imaging, and CSF cytology, as well as strong suspicion from the known malignancy. Our medical decision was based on several coordinated diagnostic criteria: clinical signs/symptoms consistent with leptomeningeal involvement; CSF findings suggestive of neoplastic meningitis; neuroimaging (MRI with gadolinium contrast); and MEITL as the known primary malignancy with similarly described cellularity.

When found in similar situations, especially if CSF flow cytometry is not available, clinicians should consider all differential diagnosis, for example primary CNS lymphoma (this is often of B-cell origin, occurring many times in elderly or immunocompromised patients and appearing on MRI as a dense, homogenous, often ring or solid enhancement).

The tumor cells of MEITL have an immunohistochemical panel revealing positivity for CD56 and cytotoxic T-cell markers, like extra-nodal NK/T-cell lymphoma [18].

Yuma Nato et al. [10] decided to treat five patients with HSCT-4 with autologous HSCT and 1 patient with allogenic HSCT. The results indicated that high-doses of chemotherapy–methotrexate, procarbasine, and vincristine with autologous HSCT or allogenic HSCT may be promising approaches for MEITL therapeutic management.

Some of the recommended chemotherapy regimens are CHOP—cyclophospamide, doxorubicin, vincristine, etoposide, prednisone; CHOEP—cyclophosphamide, doxorubicin, vincristine, etoposide, prednisone; and COP—cyclophosphamide, vincristine, prednisone, Ro-romidepsin. The latter can be associated with stem cell transplantation and radiotherapy.

## 5. Conclusions

MEITL is a rare and aggressive type of intestinal T-cell lymphoma. Its main features include monomorphism (tumor cells of relatively uniform shape and size), lymphoepithelial lesions, jejuno-ileal involvement (rarely colon or stomach), and immunohistochemical positive panel for CD3, CD8, CD56 and TIA-1, but negative for CD5 or CD4.

A positive diagnosis requires complex investigations, such as histopathological and immunohistochemical analysis, blood tests, with or without bone marrow aspirate, imaging studies such as -CT, PET-CT, and (for leptomeningeal secondary dissemination), whole-brain MRI with contrast, and CSF examination (biochemistry, cytology, flow cytometry).

The case we presented is a rare situation of leptomeningeal secondary dissemination of MEITL, associating also urothelial carcinoma. We were not able to identify a similar case in the available literature. The inflammatory biological syndrome and intestinal tumor with suggestive histopathology, corroborated by the CSF findings, contributed to the positive diagnosis of our patient. The diagnosis was particularly difficult due to the lack of CSF flow cytometry, which can often be the case in many centers.

## Figures and Tables

**Table 1:** CSF analysis details.

RELEVANT CSF ANALYSIS FINDINGS	COMMENTS ON THE INTERPRETATION OF RESULTS
Albumin levels—99 mg/dL (reference interval 10–30 mg/dL)	Indicative for blood–brain barrier impairment; significantly increased protein may suggest CNS tuberculosis, also fungal, or neoplastic meningitis
Chlorides 115 mmol/L (reference interval 118–132 mmol/L)	Normal level
Glycorrhachia—50 mg/dL (reference interval 50–80 mg/dL)	Normal level
Pandy +++ reaction	Strongly positive—indicates significant elevation in globulins (protein inflammation)
Cell Count: 175 elements/mmc, with 80% monomorphic lymphocytes (normal interval 175 elements/mm^3^)	Pleocytosis with lymphocytic predominance may suggests chronic inflammation, such as tuberculosis, fungal, viral, or neoplastic meningitis.
Negative bacterial cultures	No bacterial growth—argues against acute bacterial meningitis
Opalescent appearance, xanthochrome liquid	Suggests prior bleeding (xanthochromia) and protein elevation

## Data Availability

The original contributions presented in this study are included in the article. Further inquiries can be directed to the corresponding author.

## Abbreviations

The following abbreviations are used in this manuscript:

ALS	amyotrophic lateral sclerosis
CD	lymphocyte-specific surface makers
CT	computed tomography
DCRT	definite chemoradiotherapy
EATL	enteropathy-associated T-cell lymphoma
ESR	erythrocytes sedimentation rate
HSCT	hematopoietic stem cell transplantation
MRI	magnetic resonance imaging
MEITL	monomorphic epitheliotropic intestinal T-cell lymphoma
PET-CT	positron emission tomography-computed tomography
WHO	World Health Organization

## References

[1] Huang L., Yang H., Yang Y., Yu F., Zhao Y., Ye X., Zheng W., Sun J., Zhang E., Cai Z. (2023). Hodgkin Lymphomas and T/NK cell lymphomas: Clinical and epidemiological. Blood.

[2] Chen C., Gong Y., Yang Y., Xia Q., Rao Q., Shao Y., Zhu L., Zhang J., Li X., Ji P. (2021). Clinicopathological and molecular genomic features of monomorphic epitheliotropic intestinal T-cell lymphoma in the Chinese population: A study of 20 cases. Diagn. Pathol..

[3] Haddad P.A., Dadi N.C. (2020). Clinicopathologic determinations of survival in monomorphic epitheliotropic intestinal T-cell lymphoma (MEITL): Analysis of a pooled database. Blood.

[4] Veloza L., Cavalieri D., Missiaglia E., Ledoux-Pilon A., Bisig B., Pereira B., Bonnet C., Poullot E., Quintanilla-Martinez L., Dubois R. (2023). Monomorphic epit heliotropic intestinal T-cell lymphoma comprises morphologic and genomic heterogeneity impact outcome. Haematologica.

[5] Kucuk C., Jiang B., Hu X., Zhang W., Chan J.K., Xiao W., Lack N., Alkan C., Williams J.C., Avery K.N. (2015). Activating mutations of STAT5B and STAT3 in lymphomas derived from gammadelta-T or NK cells. Nat. Commun..

[6] Takeshita M., Nakamura S., Kikuma K., Nakayama Y., Nimura S., Yao T., Urabe S., Ogawara S., Yonemasu H., Matsushita Y. (2011). Pathological and immunohistological findings and genetic abberations of intestinal entheropathy-associated T-cell lymphoma in Japan. Histopathology.

[7] Hamada Y., Tanaka K., Nakamura M. (2019). Unusual case of abdominal fullness in a middle-age woman. Gastroenterology.

[8] Huang D., Lim J.Q., Cheah D.M.Z., Kahliab K.H.B.M., Laurensia Y., Pang J.W.L., Wong E.K.Y., Chia B.K.H., Goh J., Zhang X. (2020). Whole-genome sequencing reveals potent therapeutic strategy for monomorphic ephiteliotropic intestinal T-cell lymphoma. Blood Adv..

[9] Roberti A., Dobay M.P., Bisig B., Vallois D., Boéchat C., Lanitis E., Bouchindhomme B., Parrens M.C., Bossard C., Quintanilla-Martinez L. (2016). Type II enteropathy-associated T-cell lymphoma features a unique genomic profile with highly recurrent SETD2 alterations. Nat. Commun..

[10] Nato Y., Miyazachi K., Imai H., Nakano E., Kageyama Y., Ino K., Fujieda A., Matsumo T., Tawara I., Tanaka K. (2021). Early central nervous system relaps of monomorphic epitheliotropic intestinal T-cell lymphoma after cord blood transplantation. Int. J. Hematol..

[11] Gobbi C., Buess M., Probst A., Ruegg S., Schrami P., Herrmann R., Steck A.J., Dirnhofer S. (2003). Entheropathy-associated T-cell lymphoma with initial manifestation in CNS. Neurology.

[12] Uematsu N., Sumi M., Kaiume H., Takeda W., Kirihara T., Ueki T., Hiroshima Y., Ueno M., Ichikawa N., Watanabe M. (2018). Neurolymphomatosis duet o entheropathy-associated T-cell lymphoma clinically diagnosed by FDG-PET/CT and subsequently confirmed by autopsy. Rinsho Ketsueki.

[13] Tutt A.N., Brada M., Sampson S.A. (1997). Entheropathy associated T cell lymphoma presenting as an isolated CNS lymphoma three years after diagnosis of celiac disease: T cell receptor polymerase chain reaction studies failed to show the original entheropathy to be a clonal disorder. Gut.

[14] Shams P.N., Waldam A., Dogan A., Mackenzie J.M., Plant G.T. (2002). Ataxia in the settings of complicated entheropathy: Double jeopardy. J. Neurol. Neurosurg. Psychiatry.

[15] Defillo A., Zelensky A., Smmons B.H., Nussbaum E.S. (2012). Supratentorial metastatic enteropathy-associated T-cell lymphoma: A case report and literature review. Surg. Neurol. Int..

[16] Berman E.L., Zauber N.P., Rickert R.R., Diss T.C., Iscaacson P.G. (1998). Entheropathy-associated T-cell lymphoma with brain involvement. J. Clin. Gastroenterol..

[17] Chuah Y.Y., Tashi T., Lee Y.Y., Fu T.Y., Shih C.A. (2020). Entheropathy-associated T-cell Lymphoma (EATL) with intracranial metastasis: A rare and dismal condition. Acta Gastro-Enterol. Belg..

[18] Lungu M., Telehuz A., Voinescu D.C., Sapira V., Trifan A., Elkan E.M., Fatu A., Creanga V.Z., Polinschi M., Stoleriu G. (2021). NK/T-cell non-Hodgkin lymphoma: Case report and review of the literature. Exp. Ther. Med..

[19] Guo X., Wang Y. Monomorphic Epitheliotropic Intestinal. PathologyOutlines.com Website. https://www.pathologyoutlines.com/topic/lymphomameitl.html.

[20] Christian H., Murga-Zamalloa C.A. Primary CNS Lymphoma. PathologyOutlines.com Website. https://www.pathologyoutlines.com/topic/lymphomaprimaryCNSlymphoma.html.

